# A framework for considering the utility of models when facing tough decisions in public health: a guideline for policy-makers

**DOI:** 10.1186/s12961-022-00902-6

**Published:** 2022-10-08

**Authors:** Jason Thompson, Roderick McClure, Nick Scott, Margaret Hellard, Romesh Abeysuriya, Rajith Vidanaarachchi, John Thwaites, Jeffrey V. Lazarus, John Lavis, Susan Michie, Chris Bullen, Mikhail Prokopenko, Sheryl L. Chang, Oliver M. Cliff, Cameron Zachreson, Antony Blakely, Tim Wilson, Driss Ait Ouakrim, Vijay Sundararajan

**Affiliations:** 1 Transport, Health and Urban Designed (THUD) Research Laboratory, Melbourne School of Design, The University of Melbourne, Melbourne, Australia; 2 Centre for Human Factors and Sociotechnical Systems, The University of the Sunshine Coast, Sippy Downs, Australia; 3 Faculty of Medicine and Health, University of New England, Armidale, Australia; 4 Burnet Institute, Melbourne, Australia; 5 Monash University, Melbourne, Australia; 6 Barcelona Institute for Global Health (ISGlobal), Hospital Clinic, University of Barcelona, Barcelona, Spain; 7 Centre for Behaviour Change, University College London, London, United Kingdom; 8 National Institute for Health Innovation, The University of Auckland, Auckland, New Zealand; 9 Centre for Complex Systems, The University of Sydney, Camperdown, Australia; 10 School of Physics, The University of Sydney, Camperdown, Australia; 11 School of Computing and Information Systems, The University of Melbourne, Melbourne, Australia; 12 Sydney Institute for Infectious Diseases, The University of Sydney, Camperdown, Australia; 13 School of Population and Global Health, The University of Melbourne, Melbourne, Australia; 14 Department of Public Health, La Trobe University, Melbourne, Australia; 15 University Department of Rural Health, Faculty of Dentistry, Medicine and Health Sciences, The University of Melbourne, Melbourne, Australia; 16 McMaster Health Forum, McMaster University, Hamilton, ON Canada; 17 Department of Health Research Methods, Evidence and Impact, Faculty of Health Sciences, McMaster University, Hamilton, ON Canada

**Keywords:** Policy, Decision support, Decision-making, Public health, Modelling

## Abstract

The COVID-19 pandemic has brought the combined disciplines of public health, infectious disease and policy modelling squarely into the spotlight. Never before have decisions regarding public health measures and their impacts been such a topic of international deliberation, from the level of individuals and communities through to global leaders. Nor have models—developed at rapid pace and often in the absence of complete information—ever been so central to the decision-making process. However, after nearly 3 years of experience with modelling, policy-makers need to be more confident about which models will be most helpful to support them when taking public health decisions, and modellers need to better understand the factors that will lead to successful model adoption and utilization. We present a three-stage framework for achieving these ends.

## Introduction

Standing side by side with scientists and public health officials, leaders and policy-makers announcing difficult public health measures and social restrictions in response to surging COVID-19 cases, vaccine side-effects, or business and social pressures have been at pains to explain that they are “following the science” and/or the “advice of health and medical experts”.

The vison of science and medicine embodied here is of its popular conceptualization: white coats, laboratories, stethoscopes, test tubes, highly controlled experiments and observation. Yet in reality, the science driving much of the decision-making likely looks very different. The modelling aspects of the scientific effort can resemble a dispersed network of mathematical and/or computational modellers drawing in multiple sources of data and expertise from across the public and academic realm, and deploying software models on remote, high-performance computing clusters. These efforts utilize information from several quarters (e.g. laboratory science, epidemiological evidence and behavioural science) and then consolidate and integrate these data alongside expert judgement and abstractions of social norms, dynamics, networks and structures, interactions, cognition and patterns of movement in efforts to generate an overall representation of the world, its mechanics and likely trajectory under different policy settings and conditions.

These representations are often agent-based models (ABMs) that mirror fine-grained artificial societies. Others may be system dynamics models, compartmental models, discrete event simulation models or other associated mathematical representations. Each approach has strengths, weaknesses and levels of sophistication and complexity [1].

We draw a somewhat artificial distinction [2] between mathematical and computational models here by describing mathematical models as those typically used to describe patterns in observed data and understand relationships between variables that may hold true into the future. By contrast, computational models are developed more to mirror dynamic processes and mechanisms presumed to drive system outcomes over time. Dependent upon the point and depth to which policy-makers might wish to observe, predict, forecast or intervene in a system, both mathematical and computational models can be effective tools for enabling understanding and exploration of complex, dynamic relationships between multiple factors that affect the operation of systems combining spatial, social, behavioural and biological processes (e.g. pandemics) [3]. They can be useful for enabling policy-makers to examine ideas and interventions, and test them within synthetic societies prior to taking real-world action [4]. So, while the COVID-19 pandemic has rapidly accelerated collaboration and progress across many research domains [5] (e.g. vaccine development), it has also produced a proliferation of computational public health models of the type intended for use by government, social care and health system managers to support better decision-making [6, 7].

However, despite their potential, some computational models (such as ABMs) have been regarded as so-called black boxes when compared with more standard compartmental models, which are not developed from a systems-thinking perspective. They can therefore fail to engage the trust of intended recipients [8]. This failure must be overcome if computational models and their potential benefits are to be embedded in regular decision-making processes, and for assisting leaders in making better-quality decisions.

### Public health modelling amid crises

In the area of public health, models can be used as a tool for structured decision-making as well as informing, analysing, explaining, speculating and planning [9]. Models enable decision-makers to consider the potential impact of different variables (e.g. vaccination rates) and policy decisions (e.g. mask mandates, lockdowns) on defined population health outcomes [6, 10–12]. Whilst the desire from the public and policy-makers is often that models be predictive, realistically, prediction at fine-scale within complex, dynamic systems is a difficult if not impossible challenge [13]. Models are therefore perhaps more usefully embraced as tools for forecasting the likely pattern of outcomes that might emerge under various conditions over time [6]. They can therefore also be used to estimate the health and economic costs and benefits of different public health interventions [14].

The use of models to support public health decision-making is also nothing new. Multiple reports, articles and guidelines lay out clear, methodical means of model development with policy-makers that include principles of collaboration, participation and iteration [15–19]. However, most of these frameworks have been created under assumptions that both the modeller and user are not facing immediate crises that preclude lengthy development and collaboration cycles (i.e. a global pandemic).

Policy-makers facing novel, urgent crises with deep uncertainty and often limited scientific understanding or training [20–23] cannot politically or ethically wait for the passing of so-called normal scientific processes and maturation of evidence before acting [4, 24]. Policy-makers engaged with the science [25, 26] will quickly realize that formal models constructed amid emerging crises need to incorporate available and/or sufficient evidence more so than providing absolute certainty [17]. This point was highlighted in the early stages of the SARS-CoV-2 pandemic by Michael Ryan, Director of WHO’s Health Emergencies Programme, who noted, “If you need to be right before you move, you will never win. Perfection is the enemy of the good when it comes to emergency management” [24].

The key consideration for policy-makers facing crises is not to discard models that cannot deliver evidence with very high certainty, but to recognize (1) features of models that indicate they will be useful, and (2) that the evidence generated by them is both timely and robust enough to be acted upon.

We therefore recommend that policy-makers undertake a “rapid appraisal” of models made available to them. In doing this, we recommend they consider three elements of model utility:

1. instrumental utility, *taking into account model*
  - inputs
  - mechanisms, and
  - outputs;
2. conceptual utility; and
3. political utility [27].

We step through these elements below.

Instrumental utility requires the model to produce evidence that adequately answers questions posed of it by policy-makers—that is, it is “fit for purpose”. Policy-makers should feel as though they are “in” the model and can “drive” it by manipulating its policy levers and experimenting with scenarios. The model should look at a problem and attempt to solve it from the policy-maker’s perspective.

Evidence that has instrumental utility can be used to adjust or inform policy decisions facing governments and administrators (i.e. the evidence purports to show what policy levers are available, how and when those policies could be enacted, and what the consequences might be). This is the typical technical goal of modelling teams. A model that has instrumental utility is calibrated, valid and robust, and provides guidance that is as clear and accurate as possible given the uncertainty inherent in the crisis. As far as possible, the model should meet formal criteria for quality of theory, realism and mechanics, and objectivity as set out by the discipline(s) that contributed to its structure. It should be transparent, reproducible and able to generate results within an adequately short time frame for it to be used in crisis planning. It should demonstrate both internal and external validity.

The technical robustness of the model’s construction is just as valuable as its theoretical robustness, since minor technical errors can propagate into more significant errors, resulting in incorrect advice that, in turn, may lead to nonoptimal policy decisions. A model formally defined according to the criteria set out by the contributing discipline(s) should be implemented with formal testing and verification methods where possible [28]. Transparent model-checking [29] using formal methods is crucial in maintaining a model’s instrumental utility. For example, formal validation and verification used in electronic voting systems help maintain public trust and policy-maker support [30].

**Fig. 1:**
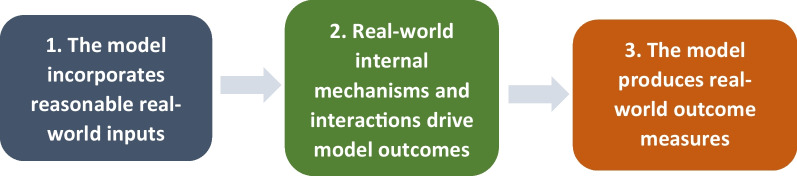
Qualities policy-makers should look for in models used to assist decision-making

To assess the model’s instrumental utility, the user should broadly compare three elements of what they know of the real world with what they understand of the presented model world. They should ask what the concordance is between the real world and model inputs (e.g. features and elements being included in the model), mechanisms (i.e. the *way* the model works by combining model inputs and answers to questions of “what is going on here?”) and outputs (the range of outcomes generated by the model that describe its performance) (see Fig. 1). If there is a high degree of agreement across all three areas, the model is more likely to be a faithful representation of reality and its outcomes more likely to be trustworthy. Taking these elements individually, it is self-evident that poor input data will result in poor output data even if a model’s mechanics are sound. Poor model mechanics will turn even good input data into poor output data or misrepresent the *way* (i.e. policies) good policy outcomes might be achieved [16]. And poorly specified or limited outcome data (e.g. not appreciating unintended effects of policy decisions) will not provide policy-makers with adequate insight into the required range of outcomes they need (e.g. health and economic considerations of policy choices).

*Conceptual utility* is the model’s capacity to be effectively communicated, thereby convincing the public, policy-makers, advisors and co-decision-makers that the evidence produced by the model is robust and explainable, makes sense and can be trusted. Evidence with conceptual utility can influence the perceptions of policy-makers, public servants and the general public around the need for policy action (i.e. the evidence is stark, easily communicated, transparent and convincing). The model and the evidence it provides should have so-called face validity for the public and those around the decision-making table. Model transparency as described above is important, as are the qualifications, expertise, backgrounds and track records of the model producers. The team or individuals who created the model should be known, credible and respected by their peers. They should be perceived as independent, free from conflicts of interest and/or from respected independent institutions. The model should broadly agree with evidence from other sources, but also generate occasional surprising but explainable moments of new insight for users. All in all, the model must deliver valuable insight that is not possible without it, and must be seen to provide credible, transparent evidence that can be trusted and defended.

For models to have *political utility*, their instrumental and conceptual utility must be strong enough to support defensible policy actions that will be supported by the community over extended time. Political utility enables the implications of the model to be actively and successfully woven into policy-making and desired actions of government. A model that does not have conceptual utility will not have political utility. A model that does not have instrumental utility will be found wanting through inaccuracy, so will also lose conceptual and eventually political utility. Once credibility is lost, trust in models will then be difficult to recover, and future efforts may be met with increased scepticism, leading to reluctance on the part of the community to comply with policy-makers’ directives.

**Fig. 2:**
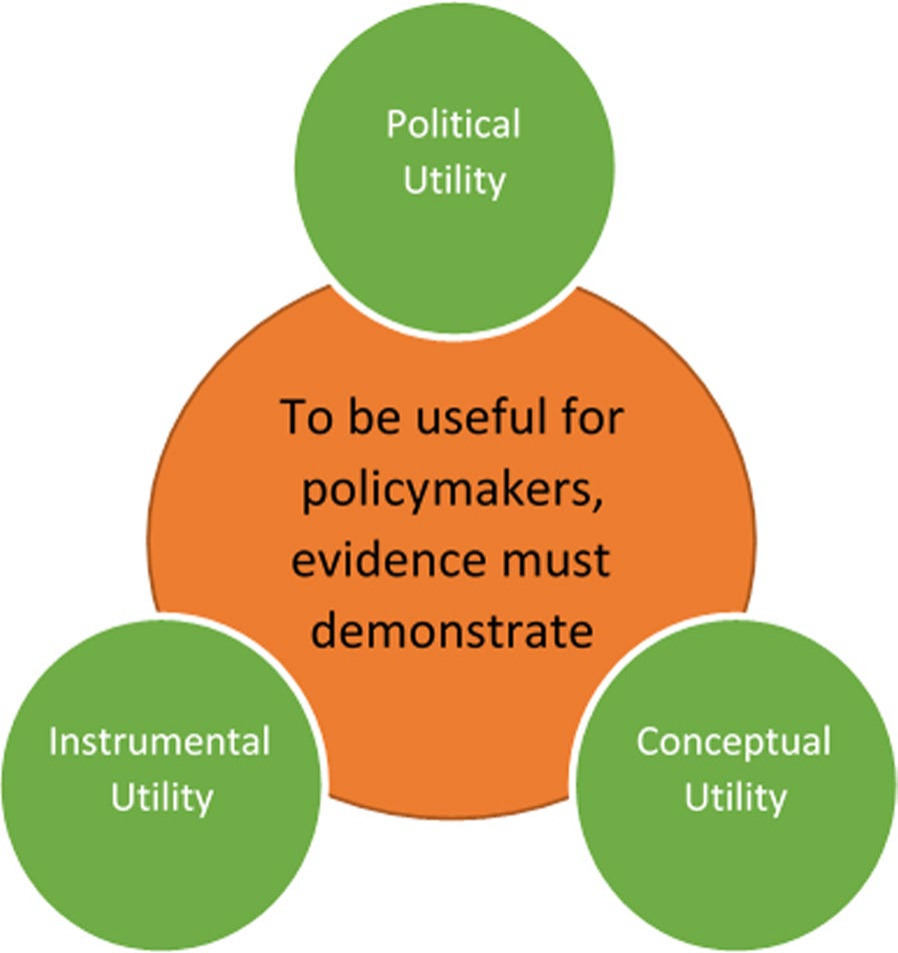
Three elements of instrumental, conceptual and political utility that assist models in being useful for policy-makers

Only when these three interdependent forms of utility derived from the model’s outputs are satisfied (see Fig. 2) are windows of opportunity [31] likely to open for scientific evidence derived through models to successfully integrate with public policy-making.

Using these criteria, Table 1 provides a set of guidelines that policy-makers can use to evaluate whether any presented model approaches the thresholds for use in their local context.

**Table 1:** Features of models that provide utility to policy-makers in times of crisis

Utility element	Model characteristics	
Instrumental utility (the model works and is fit for purpose)	Data inputs •Data inputs used to drive the model are robust, verified (where possible) and adequate for the purpose of model outputs •The model inputs include ranges of uncertainty •The model is of appropriate scale to capture the problem faced by decision-makers •The model strikes an adequate balance between sophistication and simplicity (i.e. abstraction) across model elements and representation of phenomena •The model appreciates differences between individuals and groups (e.g. age vulnerability differences) that are important for understanding significant differences in outcomes at the population level •The model has been specified correctly across variables of importance to model outcomes Mechanics •The mechanics of the model are formally defined and explainable at a technical level by its authors (i.e. it is not a “black box”) •The model has had interdisciplinary input and/or does not come from a single person or source •The model structure and/or its components have been formally tested and verified, as well as by other independent experts •The model is open—i.e. its authors are happy to share its mechanics with the world and have those open to scrutiny •Interactions of variables and features in the model are reflective of—or analogous to—real-world interactions •Understanding the mechanics of the model enables insight into *how* outcomes and output data are generated •Data and assumptions are adjusted iteratively to take account of new evidence Data outputs •The model estimates outputs over time frames of relevance to policy-makers •The model provides clear policy direction and guidance based on agreed system performance metrics (e.g. health, economic/financial costs, public acceptance of measures) [32] •The model appreciates trade-offs of implemented policies across associated domains •The model outputs can be validated against historical data •The model outputs include ranges of uncertainty •The modellers can explain which variables have the greatest influence on model outcomes and overall system performance (i.e. can provide sensitivity analyses where appropriate) •The authors of the model can explain their confidence in the outputs of the model, which is a separate consideration from the confidence intervals produced by the model outputs •The model produces results that are broadly consistent with other like models or representations but also adds unique insight that other models might not General •The model framework, including assumptions and outcome measures, has been developed in collaboration with policy-makers (as far as possible) •The model is fast enough to provide guidance in the time frame required by policy-makers •The model is of adequate scope to capture and reflect the problem faced by policy-makers •The model is being used for the purposes it was designed •The authors can clearly articulate what the model is missing, what level of detail it cannot capture, what it can’t tell the user, and what it should not be used for, and the possibility of the model being affected by “off-model” events	
Conceptual utility (the model is understood)	•The model is transparent—each aspect of it is explainable in plain language to a naïve audience—it is not a “black box” that neither the model authors nor outside experts can explain •The model looks and sounds credible to a naïve audience and/or the audience (e.g. general public) who will be subject to its recommendations •The model authors and contributors are suitably qualified and experienced •The model authors are independent and/or there is no apparent conflict of interest •The model authors’ institution is suitably qualified and experienced, and their institution is independent from political decision-making •The model appeals to common sense but is sophisticated enough to extend the boundaries of people’s ability to conceptualize multiple future scenarios •The model results are at times surprising but remain logical and explainable when surprising results emerge, demonstrating insight that might not otherwise have been gained through informal, implicit modelling •Model results are presented plainly and implications are self-evident •Model authors and/or their institution are presentable and can defend the validity of their work to the public •The model has a public interface and/or can be manipulated by the public and/or other end-users to aid understanding •The range of uncertainty in estimates is made clear to policy-makers	
Political utility (the policy implications of the model are supported)	•The implications of the model can be woven into an acceptable, consistent political narrative by policy-makers •The model has adequate instrumental utility as described above •The model has adequate conceptual utility as described above •Decision-makers (ministers, public servants and health authorities) have input into the modelling and its assumptions as early as possible in the build process •The model outputs and recommendations are accepted as robust by policy-makers •If policy-makers plan to use the model and/or its authors to prosecute public health initiatives, they are prepared to implement recommendations as per the model design and/or clearly articulate which aspects of the model they are taking recommendations from •The model is/is proving to be accurate •The model authors are/are proving to be reliable communicators and support its use in the way it is being used •The relationship between model authors and policy-makers remains collaborative and productive •The public continue to support the policy implications of the model’s findings	

## Conclusions

Computational modelling in public health can be an extremely low-cost, high-value exercise. In crisis situations where public health is at stake, models can have even greater utility. Despite this, computational models have not progressed far beyond the realm of “toys” in the minds of some policy-makers [8]. Worse, poor models or poor experiences with models—including overpromising predictive power—can result in the entire field being dismissed by policy-makers and the public as confusing, contradictory and untrustworthy [33].

To help alleviate this problem, we present a framework that asks both model developers and policy-makers to evaluate the utility of models across three related dimensions. This framework asks more from model developers who intend their work to be used for decision support in public health. We ask model developers to ensure their efforts are geared towards use by model users: that their models are fit for purpose, that they are transparent and comparable, and that they achieve this for the purposes of adoption and application in the real world. We suggest that to be adopted, they need to demonstrate qualities of instrumental, conceptual and political utility.

Computational modelling has the potential to act as a consolidating discipline that sits at the interface of science, public health research and public policy across the world. Greater integration between model developers and end-users on what creates a useful model is likely to enhance the understanding and utilization of computational models, as well as the models themselves.

## Data Availability

The datasets during and/or analysed during the current study available from the corresponding author on reasonable request.
